# Airway Inflammatory/Immune Responses in COPD and Cystic Fibrosis

**DOI:** 10.1155/2018/7280747

**Published:** 2018-05-02

**Authors:** Virginia De Rose, Pierre-Régis Burgel, Amit Gaggar, Catherine Greene

**Affiliations:** ^1^Department of Clinical and Biological Sciences, University of Torino, A.O.U. S.Luigi Gonzaga, Regione Gonzole 10, 10043 Orbassano, Torino, Italy; ^2^Paris Descartes University and Cochin Hospital, Assistance Publique-Hôpitaux de Paris, Paris, France; ^3^University of Alabama at Birmingham, Birmingham, AL, USA; ^4^Lung Biology Group, Department of Clinical Microbiology, Royal College of Surgeons in Ireland, Education and Research Centre, Beaumont Hospital, Dublin 9, Ireland

Cystic fibrosis (CF) is a genetic disease caused by mutations in the cystic fibrosis transmembrane conductance regulator (CFTR) gene and remains one of the most common fatal hereditary disorders worldwide. Although CF is a complex multiorgan disease, morbidity and mortality are mainly determined by progressive chronic obstructive lung disease.

COPD is a major global health problem. The disease is caused by both genetic and environmental factors; among these, cigarette smoking (CS) is the main risk factor and is associated with a marked oxidative stress burden and a persistent airway inflammatory response.

Although the etiology and the pathophysiology of these diseases are different, they share key phenotypical features, including reduced mucociliary clearance, airway mucus obstruction especially in small airways, chronic neutrophilic airway inflammation, and chronic/recurrent bacterial infections. In both diseases, a persistent high-intensity inflammation, driven by continuous neutrophil recruitment, leads to permanent structural damage of the airways and progressive airflow obstruction.

Several defective inflammatory/immune responses have been linked to CFTR deficiency including innate and acquired immunity dysregulation, cell membrane lipid abnormalities, and various transcription factor-signaling defects. During the past few years, several *in vitro* and *in vivo* studies have shown that CS decreases CFTR expression and function and induces an acquired CFTR dysfunction in patients with a normal CFTR genotype. Thus, it is possible that common mechanisms may contribute to the chronic nonresolving inflammation and the altered immune responses in both CF and COPD. These mechanisms however are still poorly defined.

This special issue aims to contribute to knowledge of these mechanisms and to provide a better understanding of altered inflammatory/immune responses in both CF and COPD as well as a roadmap for potential novel therapeutic approaches relevant to both diseases. We hope this issue may be a useful reference to all readers and investigators interested in potential mechanistic links and common therapeutic targets in CF and COPD and that it may be an incentive for further investigations in this field.

Airway epithelial cells are among the first sites of contact for pathogens and other noxious environmental irritants and play a critical role in maintaining normal airway functions as well as in modulating inflammatory/immune responses in the airways. Relevant molecular and morphologic changes occur in the airway epithelium in both CF and COPD. In their review, V. De Rose et al. addressed the evidence for a critical role of dysfunctional airway epithelium in impaired local defences, altered immune responses, chronic airway inflammation, and remodelling in CF and COPD, highlighting the common mechanisms involved in epithelial dysfunction as well as the similarities and differences in the two diseases. They discuss the *in vitro* and *in vivo* findings showing that CS induces an acquired CFTR dysfunction in patients with COPD, reducing the expression and/or function of the protein; they highlight that this CFTR dysfunction is involved in most of the pathogenetic pathways common to both COPD and CF and may represent a potential target for the development of novel therapeutic approaches in COPD.

M. Stolarczyk and B. J. Scholte reviewed the role of the EGFR/ADAM17 axis in the development of chronic lung disease in CF and COPD. They discuss the evidence suggesting that the ADAM17/EGFR axis and downstream regulatory pathways are hyperactive in both diseases. The enhanced ADAM17/EGFR signaling may contribute to inflammation, epithelial metaplasia, and fibroblast and smooth muscle activation, as well as tissue remodelling observed in CF and COPD lung disease. They also discuss a possible mechanistic link between EGFR/ADAM17 activity, CF, and COPD, suggesting that the genetic CFTR defect in CF and the CS-induced CFTR dysfunction in COPD interfere with glutathione transport in the airways, enhancing oxidative stress, which would activate the ADAM17/EGFR axis.

In the context of airway inflammation and mucosal immunity, M. Puccetti et al. have reviewed the role of the aryl hydrocarbon receptor (AhR) in COPD and CF lung disease. They discuss the effects of this receptor on the immunological status of the gastrointestinal and respiratory tracts and highlight its relevance in establishing and maintaining signaling networks which facilitate host/microbe homeostasis at the mucosal interface. They also analyze the evidence suggesting that changes in AhR expression and function may be a risk factor for COPD and other lung inflammatory diseases in smokers and that the AhR status could be dysregulated in smokers. Finally, they also discuss the possible therapeutic use of AhR ligands in cystic fibrosis.

A common feature shared by CF and COPD is the increased susceptibility to respiratory infections; recurrent infectious exacerbations significantly contribute to morbidity and mortality in both diseases. To better understand the mechanisms of recurrent exacerbations in COPD, G. Pehote et al. investigated the mechanisms of CS-induced impairment of bacterial phagocytosis in this disease and showed that an autophagy defect mediated by CS is a critical mechanism involved in the impairment of phagocytosis that may account for recurrent exacerbations in COPD. An autophagy defect has also been described in CF and suggested to underlie the increased susceptibility to infections with certain microbes in this disease; thus, the study of G. Pehote et al. suggests additional common mechanisms responsible for the increased susceptibility to respiratory infections in COPD and CF. The findings of this study also suggest the therapeutic potential of autophagy-inducing drugs, with antioxidant characteristics, in restoring CS-impaired phagocytosis in COPD and other chronic airway inflammatory diseases, such as CF.

A genuine microbiota resides in the lung, which emanates from colonization by the oropharyngeal microbiota. Changes in the oropharyngeal microbiota might be the source of dysbiosis observed in the lower airways in patients suffering from chronic airway inflammatory diseases, including COPD, asthma, and CF. S. Boutin et al. analyzed whether differences occur in the throat microbiota of children with asthma and CF in comparison to that of healthy children and reported that the microbiota in these three populations shows high levels of similarities, revealing the existence of a core microbiome. However, in the CF group, a decrease in both diversity and total bacterial load in the throat microbiota was observed in comparison to asthmatic and control children, whereas, on the contrary, a significant increase was found in typical pathogens like *Pseudomonas* and *Staphylococcus* and the atypical pathogen *Phyllobacterium*, which is consistent with the impaired host defences associated with CFTR dysfunction in the CF airways.

Toll-like receptors (TLRs) expressed on the airway epithelium respond to infection or tissue damage by sensing local microbial and host-derived factors; TLRs recognize LPS which activates intracellular molecules such as IL-1 receptor-associated kinases (IRAKs) leading to overproduction of proinflammatory cytokines. IRAK-M, a negative regulator of TLR-mediated NFkB activation, is expressed in both airway epithelial cells and monocytes/macrophages in healthy lungs. H. Gong et al., using IRAK-M KO mice, studied the effects of IRAK-M deficiency on CS-induced airway inflammation under acute or subacute conditions. They showed that IRAK-M has distinctive effects on airway inflammation and influences the Treg/Th17 balance and expression of costimulatory molecules by dendritic cells and macrophages, depending on the duration and intensity of the stimulus. In fact, whereas upon short-term CS exposure IRAK-M provided airway protection, it played a proinflammatory role in airway pathology upon subacute CS exposure. As TLRs largely contribute to chronic airway inflammation also in CF lung disease, it would be interesting in the future to investigate the effect of IRAK-M on CF airway inflammation.

Increasing evidence support a crucial role of long noncoding RNAs (lncRNAs) in controlling gene expression; furthermore, it has been shown that lncRNAs play important roles in biological and pathological processes and are dysregulated in various human diseases, including CF and COPD. X. Qu et al. evaluated lncRNAs and mRNA expression profiles of peripheral blood mononuclear cells (PBMCs) from healthy nonsmokers, smokers without airflow limitation, and COPD patients to determine if lncRNA differential expression may be linked to dysregulated mRNA expression relevant to COPD pathogenesis. They identified 158 differentially expressed lncRNAs in PBMCs from COPD patients compared with smokers without airflow limitation; they further analyzed the regulation network between lncRNAs and mRNAs, where the genes CXCL16, HMOX1, SLA2, and SIGLEC14 were predicted to be regulated by certain lncRNAs. Their study may provide clues for further studies targeting lncRNAs to control inflammation in COPD.

In summary, the articles presented in the present special issue constitute a contribution to the idea that COPD and CF share some clinical and pathophysiological aspects and that progress in the understanding of rare diseases (e.g., cystic fibrosis) could contribute to the development of novel approaches for more common diseases (e.g., COPD and bronchiectasis).

